# *Linum corymbulosum* Protects Rats against CCl_4_-Induced Hepatic Injuries through Modulation of an Unfolded Protein Response Pathway and Pro-Inflammatory Intermediates

**DOI:** 10.3390/molecules28052257

**Published:** 2023-02-28

**Authors:** Riffat Batool, Muhammad Rashid Khan, Muhammad Umar Ijaz, Irum Naz, Afsheen Batool, Saima Ali, Zartash Zahra, Safia Gul, Mohammad N. Uddin, Mohsin Kazi, Raees Khan

**Affiliations:** 1 Directorate of BASR, Allama Iqbal Open University, Islamabad 44310, Pakistan; 2 Department of Biochemistry, Faculty of Biological Sciences, Quaid-i-Azam University, Islamabad 45320, Pakistan; 3 Department of Zoology, Wildlife and Fisheries, University of Agriculture, Faisalabad 38040, Pakistan; 4 Department of Biochemistry, Institute of Biochemistry, Biotechnology and Bioinformatics, Faculty of Chemical and Biological Sciences, The Islamia University of Bahawalpur, Bahawalpur 63100, Pakistan; 5 Faculty RMU & Allied Hospitals, Rawalpindi Medical University and Allied Hospital, Rawalpindi 46000, Pakistan; 6 Gujrat Institute of Management Sciences, Peer Mehar Ali Shah Arid Agriculture University, Gujrat 50700, Pakistan; 7 Department of Botany, Sardar Bahadur Khan Women’s University Quetta, Quetta 87300, Pakistan; 8 College of Pharmacy, Mercer University, 3001 Mercer University Drive, Atlanta, GA 30341, USA; 9 Department of Pharmaceutics, College of Pharmacy, King Saud University, Riyadh 11451, Saudi Arabia; 10 Department of Plant Sciences, Faculty of Biological Sciences, Quaid-i-Azam University, Islamabad 45320, Pakistan

**Keywords:** *Linum corymbulosum*, hepatic markers, antioxidant, ER stress, pro-inflammatory intermediates

## Abstract

Liver fibrosis is a major pathological feature of chronic liver disease and effective therapies are limited at present. The present study focuses on the hepatoprotective potential of *L*. *corymbulosum* against carbon tetrachloride (CCl_4_)-induced liver damage in rats. Analysis of *Linum corymbulosum* methanol extract (LCM) using high-performance liquid chromatography (HPLC) revealed the presence of rutin, apigenin, catechin, caffeic acid and myricetin. CCl_4_ administration lowered (*p* < 0.01) the activities of antioxidant enzymes and reduced glutathione (GSH) content as well as soluble proteins, whereas the concentration of H_2_O_2_, nitrite and thiobarbituric acid reactive substances was higher in hepatic samples. In serum, the level of hepatic markers and total bilirubin was elevated followed by CCl_4_ administration. The expression of glucose-regulated protein (GRP78), x-box binding protein-1 total (XBP-1 t), x-box binding protein-1 spliced (XBP-1 s), x-box binding protein-1 unspliced (XBP-1 u) and glutamate–cysteine ligase catalytic subunit (GCLC) was enhanced in CCl_4_-administered rats. Similarly, the expression of tumor necrosis factor-α (TNF-α), interleukin-6 (IL-6) and monocyte chemo attractant protein-1 (MCP-1) was strongly increased with CCl_4_ administration to rats. Co-administration of LCM along with CCl_4_ to rats lowered (*p* < 0.05) the expression of the above genes. Histopathology of the liver showed hepatocyte injury, leukocyte infiltration and damaged central lobules in CCl_4_-treated rats. However, LCM administration to CCl_4_-intoxicated rats restored the altered parameters towards the levels of control rats. These outcomes indicate the existence of antioxidant and anti-inflammatory constituents in the methanol extract of *L. corymbulosum*.

## 1. Introduction

Chronic liver disease is a progressive destruction and regeneration of liver tissue leading to liver fibrosis and cirrhosis. It represents a major health problem and a cause of morbidity and mortality [1]. The liver has been designated a supremely elusive organ concerning peroxidative impairments, as it is rich in oxidizable elements. Metabolism of CCl_4_ in the liver induces lipid peroxidation, hepatocyte necrosis and demolition of membranes with ensuing leakage of essential enzymes into the bloodstream [2]. A single dose of CCl_4_ can strain the rat liver with oxidative stress causing severe steatosis and centrilobular necrosis. Trichloromethyl (•CCl_3_) and peroxy trichloromethyl (•OOCCl_3_) radicals, produced as a result of metabolic activation of CCl_4_ by cytochrome P_450_, cause initiation of lipid peroxidation, which is the main cause of liver and kidney damage. Hence, oxidative stress studies can be performed using a CCl_4_-induced toxicity protocol that provides a full pathological environment via generating a blend of free radicals [3].

The endoplasmic reticulum (ER), a cytoplasmic organelle, is responsible for protein maturation, protein modifications and the preservation of the homeostasis of intracellular calcium. The accumulation of unfolded proteins and an imbalance in the calcium homeostasis might disrupt ER functionality, leading to ER stress. ER stress triggers three ER membrane proteins involved in induction of the adaptive immune response: the serine/threonine kinase PKR-like ER kinase (PERK), the inositol-requiring enzyme 1 (IRE1) and the activating transcription factor (ATF6). When ER stress is too strong to restore homeostasis, CHOP activation prompts ER stress-linked apoptosis to eradicate impaired cells. ER stress is associated with many ailments such as diabetes, obesity, neurodegenerative disorders and inflammatory ailments. It also provides a target for anti-inflammatory mediators [4].

Inflammation is an important contributing factor to illness and death, and it usually occurs in nearly all diseases. Cytokines, interleukins, prostaglandins and thromboxanes are mediators that are associated with involvement in numerous metabolic and cellular events. Asthma, atherosclerosis and rheumatoid arthritis, which have high prevalence all over the world, demonstrate inflammation as a key factor that provokes or promotes these disorders [5]. As a response to inflammation, several metabolic pathways and cellular processes occur, which comprise elevation in the release of pro-inflammatory cytokines and interleukins. In the initial stage of inflammation, upregulation of interferon (INF)-γ, tumor necrosis factor-α (TNF-α), and interleukin (IL) 1, IL-6, IL-12, and IL-18 occurs, which stimulates the attraction of surplus macrophages and neutrophils leading to the production of inflammatory mediators including nitric oxide (NO) and prostaglandin (PGE_2_). Furthermore, during inflammation generation of ROS is also observed which enhances or reduces the inflammatory response along with the start of lipid peroxidation of membranes and release of injurious macromolecules. Upregulation of NFκB by increased oxidative stress enhances the synthesis of enzymes such as inducible nitric oxide synthase. (iNOS) and cyclooxygenase (COX)-2. Anti-apoptotic responses also involve NFκB [6]. Finally, in the inflamed area, the fluid leakage and leukocyte infiltration promote the formation of edema. If the innate defense of the body is unable to eradicate the stimulation of the inflammatory response this might result in many inflammatory disorders [7].

Oxidative-stress treatment with natural antioxidants is of utmost concern nowadays. Plants have been used for grazing animals for ages and for medicinal purposes in folklore and are assumed to have a possible role in health care schemes. A large amount of plant foods in a daily diet reduces mortality and morbidity risks of the abovementioned diseases [8]. Regular consumption of fresh fruits, leafy vegetables and tea supplements natural antioxidants and has an ascertained beneficial effect in preventing cardiovascular and neurodegenerative disorders [9]. Plant-produced bioactive molecules may be flavonoids (catechin, kaempferol, quercetin and naringenin), phenolic diterpenes (rosmanol, carnosic acid, carnosol, rosmarinic acids, protocatechuic, phenolic acids, gallic acid and caffeic acid) and volatile oils (menthol, eugenol, carvacrol and thymol). Amongst the prominent ones are the flavonoids [10]. Flavonoids impart anti-inflammatory, antifungal, antioxidant, antiallergic, cytotoxic, anticancer, antibacterial and hepatoprotective properties to the plant, hence confirming their worth [11].

*Linum* is the largest and most important genus of the flax family Linaceae that comprises nearly 200 taxa distributed across the temperate and warm temperate areas of Europe, Asia and America [12]. Different investigations by Baker [13] showed that many species of the genus are cultivated for their seed oils, as animal fodder and as ornaments. The seeds and leaves of many species have been utilized in traditional and modern medicine as anticancer agents, laxatives, a good source of Ω-3 fatty acids, anti-inflammatory agents, against sore throat, against burns and as angina cures [14]. *Linum corymbulosum*, a small annual herb with yellow flowers is found in Europe, the Mediterranean region, on the Canary Islands, in Africa, Afghanistan, India and Pakistan. It grows in plains and can measure up to 23 cm. The seeds are powdered and taken with water to reduce swelling; they possess an anti-inflammatory potential and are also considered an antitumoral agent [15]. In the current study, we aimed to investigate whether *L. corymbulosum* mediates antioxidant and anti-inflammatory actions against CCl_4_-induced ER stress and inflammation in rat liver.

## 2. Results

### 2.1. HPLC–DAD Analysis of L. corymbulosum Extracts

**Figure 1:**
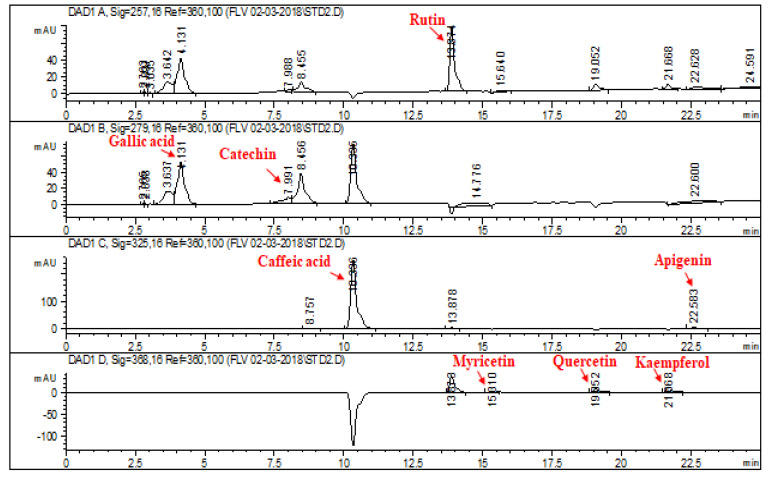
HPLC–DAD profile of reference standards for crude extracts and derived fractions of *L. corymbulosum* (LCM, LCE, LCB and LCA) at varying wavelengths. Signal 1: 257λ, signal 2: 279λ, signal 3: 325λ, signal 4; 368λ. Conditions: Mobile-phase A—ACN:MEOH:H_2_O:AA, 5:10:85:1; mobile-phase B–ACN:MEOH:AA, 40:60:1; injection volume 20 µL; flow rate 1 mL/min; Agilent RP-C8.

**Figure 2:**
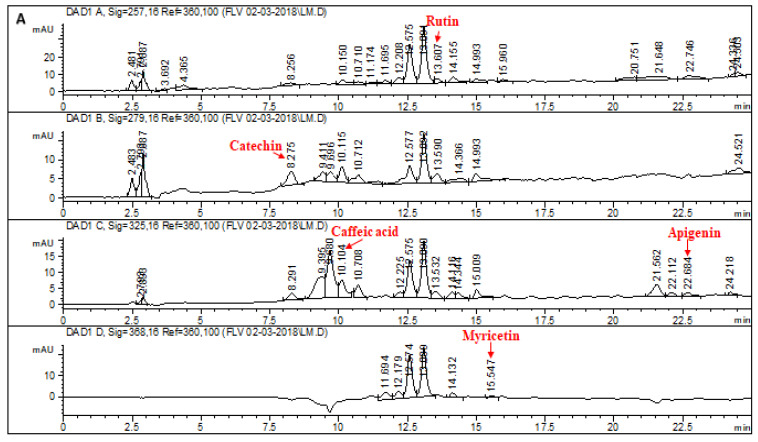

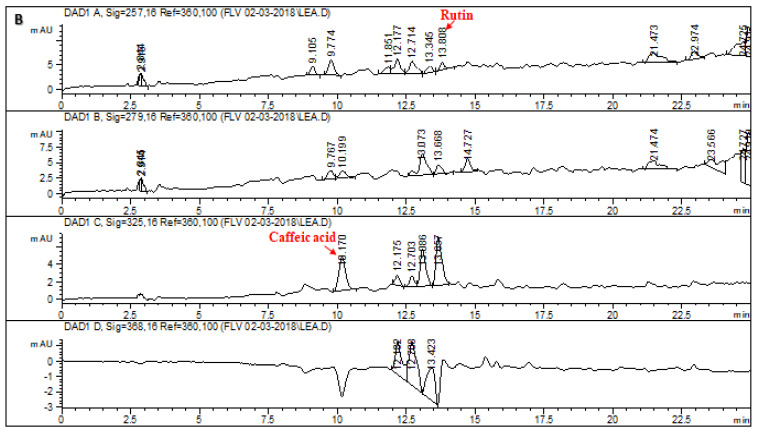

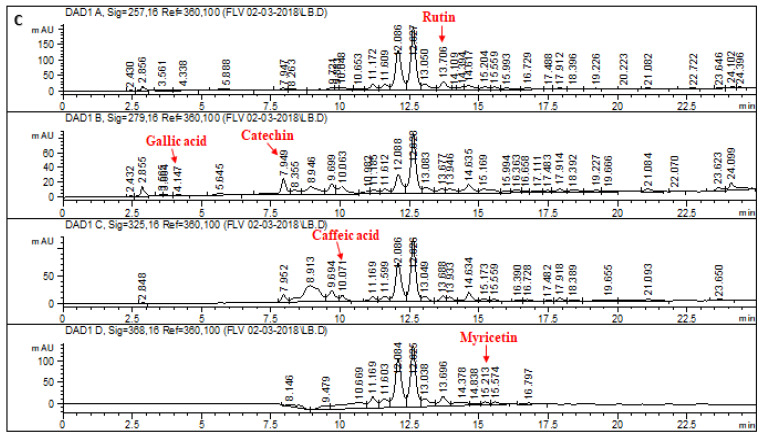

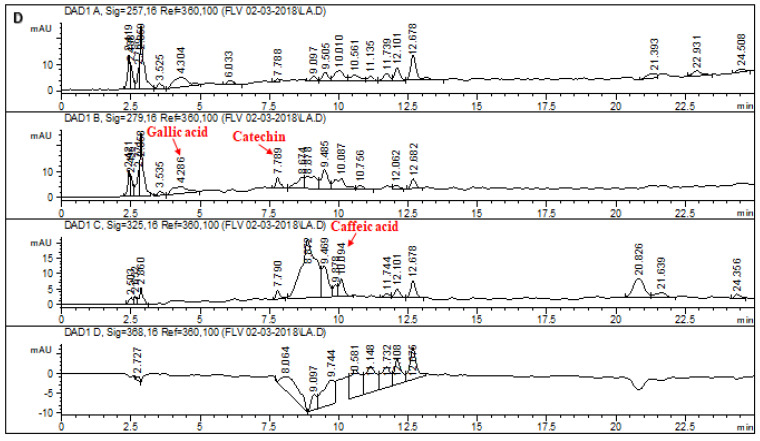
HPLC–DAD chromatogram of (**A**) LCM, (**B**) LCE, (**C**) LCB, (**D**) LCA at varying wavelengths. Signal 1: 257λ, signal 2: 279λ, signal 3: 325λ, signal 4: 368λ. Conditions: Mobile-phase A—ACN:MEOH:H_2_O:AA, 5:10:85:1; mobile-phase B—ACN:MEOH:AA, 40:60:1; injection volume 20 μL; flow rate 1 mL/min; Agilent RP-C8.

The chromatographic profile of the reference standards is depicted in Figure 1. Retention times, optimized wavelengths and regression analyses of reference flavonoids and phenolics for *L. corymbulosum* methanol extract and derived fractions are shown in Table 1. The chromatogram of the HPLC–DAD profile of LCM, LCE, LCB and LCA is illustrated in Figure 2, while the quantification of the individual fractions is summarized in Table 2. LCM exhibited the presence of rutin (1.92 μg/mg of extract), catechin (9.94 μg/mg of extract), caffeic acid (7.38 μg/mg of extract), apigenin (2.64 μg/mg of extract) and myricetin (3.37 μg/mg of extract). LCE showed the existence of only two standards, i.e., rutin (4.71 μg/mg of extract) and caffeic acid (6.21 μg/mg of extract). The HPLC profile of LCB showed the presence of five standards being used with the highest value noted for rutin (51.48 μg/mg), followed by catechin (43.7 μg/mg), myricetin (21.82 μg/mg), gallic acid (3.11 μg/mg) and caffeic acid (1.43 μg/mg). Similarly, the HPLC profile of LCA displayed the presence of catechin (7.97 μg/mg of extract), gallic acid (6.11 μg/mg) and caffeic acid (6.4 μg/mg of extract). 

### 2.2. Acute Toxicity Studies of the effect of L. corymbulosum on Hematological Parameters

The effect of LCM on various hematological parameters in rats is presented in Table 3. Administration of LCM at 2000 mg/kg bw and 4000 mg/kg bw significantly (*p* < 0.05) increased the count of white blood cells (WBC × 10^3^/mm^3^) at both doses (12.67 ± 0.26 and 13.13 ± 0.35) (*p* < 0.05) compared with the control group (6.82 ± 0.10). On the other hand, administration of 2000 mg/kg (7.10 ± 0.12) and 4000 mg/kg (7.57 ± 0.23) of LCM to rats did not induce any alteration (*p* > 0.05) in the count of red blood cells (RBC × 10^6^/mm^3^) when compared with the control group (7.33 ± 0.08). The mean hemoglobin concentration (HGB) (g/dL) of the LCM 2000 mg/kg bw group (13.3 ± 0.15) and the LCM 4000 mg/kg bw group (14.3 ± 0.69) showed no corresponding significance (*p* > 0.05) when compared with the control group (13.78 ± 0.07). The mean corpuscular volume (MCV) (fl) of the rats increased significantly (*p* < 0.05) compared with the control group following administration of LCM at 2000 mg/kg bw and 4000 mg/kg bw. A similarly significant (*p* < 0.05) increase in the number of lymphocytes (10^3^/mm^3^) and platelets (10^3^/mL) was detected in the LCM-administered low- and high-dose groups. However, there was no significant (*p* > 0.05) difference in the values of mean corpuscular hemoglobin concentration (MCHC) (g/dL) among the control (34.5 ± 0.92) and the treatment groups; 2000 mg/kg bw (37.7 ± 0.55) and 4000 mg/kg bw (39.2 ± 0.52), respectively.

### 2.3. Effect of L. corymbulosum on Body Weight and Organ Weight

The effects of LCM against CCl_4_-induced toxicity on the percent increase in body weight, as well as absolute and relative weight of the liver are presented in Table 4. Administration of CCl_4_ significantly (*p* < 0.05) reduced the percent increase in body weight while it elevated the absolute and relative weight of the liver in rats compared to the control group. Co-administration of CCl_4_-intoxicated rats with LCM increased the percent increase in body weight while a marked reduction in the absolute and relative weight of the liver was observed in these animals. Restoration of body and organ weights towards the normal control group was achieved by co-administration of 200 mg/kg bw of silymarin to CCl_4_-intoxicated rats. However, a significant (*p* < 0.05) variation in these parameters was also noted after the administration of 200 mg/kg bw and 400 mg/kg bw of LCM alone in rat.

### 2.4. Effect of L. corymbulosum on Biochemical Serum Markers

The levels of hepatic markers in the serum of all the groups are given in Table 5. The results showed a significant elevation (*p* < 0.05) in the levels of ALP, ALT, AST and bilirubin after CCl_4_ treatment and a reduction in the albumin level compared to the control group. Treatment with silymarin (200 mg/kg bw) of CCl_4_-treated rats decreased the levels of ALT, AST, ALP and bilirubin while elevating the level of albumin in serum to near those of the control group. Co-administration of LCM at low (200 mg/kg bw) and high (400 mg/kg bw) doses also efficiently restored the level of these biochemical markers towards the control group. However, the rats treated with LCM at 200 mg/kg bw and 400 mg/kg bw alone showed no significant (*p* < 0.05) difference in the concentration of ALP, ALT, AST, total bilirubin and albumin in serum compared to control rats.

### 2.5. Effect of L. corymbulosum on Hepatic Antioxidant Enzymes

The protective effects of LCM on the levels of liver antioxidant enzymes are given in Table 6. In the hepatic tissues of rats, the levels of CAT, SOD, POD and GSH were significantly (*p* < 0.05) decreased after CCl_4_ administration compared to the control group. Co-administration of LCM at low (200 mg/kg bw) and high (400 mg/kg bw) doses to CCl_4_-treated rats restored the concentration of these antioxidants towards the levels in the control group. Administration of LCM alone (low and high doses) to rats caused a significant (*p* < 0.05) decrease in the activity of the above parameters compared with control rats.

### 2.6. Effect of L. corymbulosum on Hepatic Protein, TBARS, H_2_O_2_ and Nitrite Content

Administration of CCl_4_ to rats increased the concentration of TBARS, H_2_O_2_ and nitrite in hepatic samples compared to the control group. However, protein levels were strongly decreased due to CCl_4_ toxicity (Table 7). The changes in the levels of these parameters were restored to levels comparable to the control group with co-administration of silymarin to the CCl_4_-treated group. The altered levels of these parameters induced with CCl_4_ were also restored towards the control group by co-administration of LCM at both low and high doses. Level of H_2_O_2_ and nitrite content were clearly restored at a higher dose of LCM and were close to those in the silymarin treated group.

### 2.7. Effect of L. corymbulosum on ER-Stress Markers and Inflammatory Mediators

**Figure 3:**
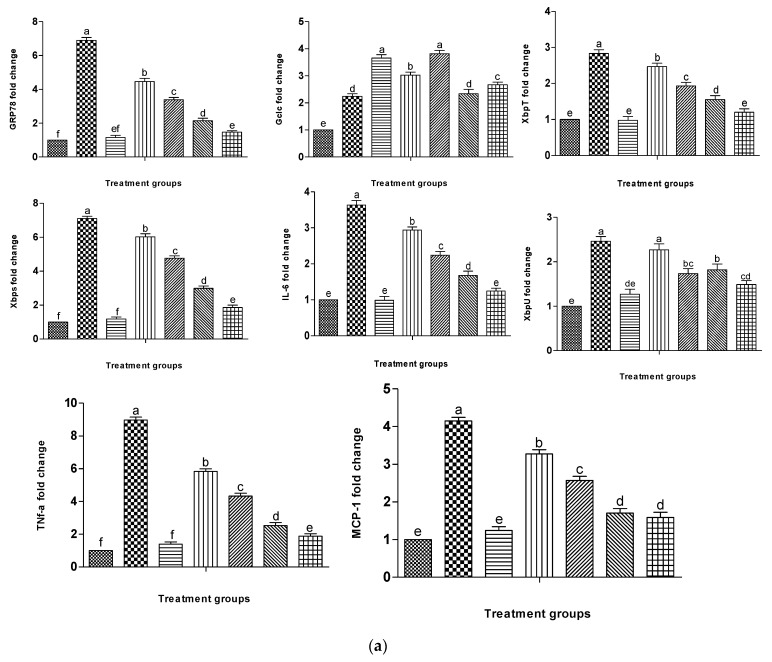

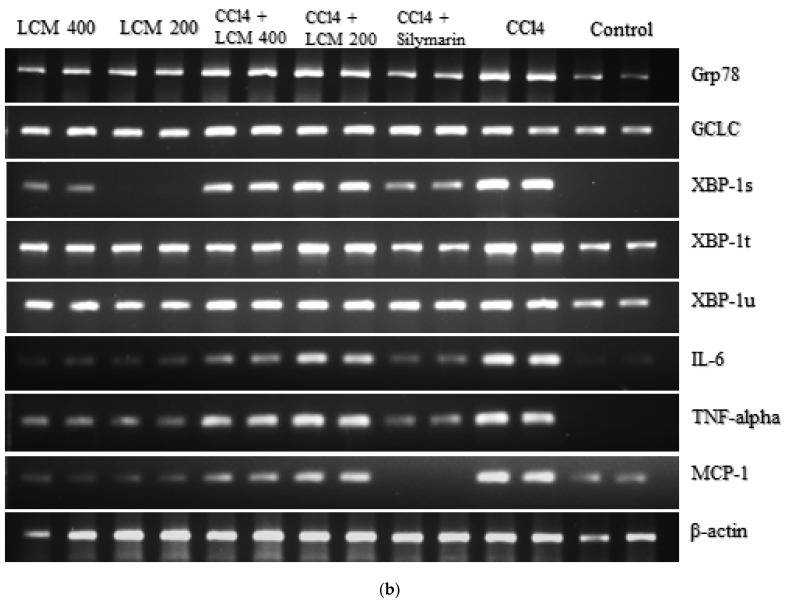
(**a**): Graphical representation of different treatments with LCM on fold changes of various genes involved in ER stress and inflammation. 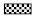 Control, 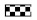 CCl_4_, 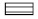 CCl_4_ + silymarin (200 mg/kg), 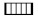 CCl_4_ + LCM (200 mg/kg), 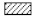 CCl_4_ + LCM (400 mg/kg), 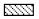 LCM (200 mg/kg), 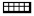 LCM (400 mg/kg). LCM; *Linum corymbulosum* methanol extract. The representation of letters (a-e) specify significance at *p* < 0.05 (**b**): Gel electrophoresis of cDNA after RT-PCR analysis.

The expression levels of mRNAs of ER stress proteins are depicted in Figure 3a and Table 8. Treatment of rats with CCl_4_ caused a significant (*p* < 0.05) increase in the expression of GRP78, XBP-1s, XBP-1t and XBP-1u compared to control animals. The expression levels of these genes were restored towards the control group by co-administration of silymarin to the CCl_4_-treated group. Similarly, co-treatment of CCl_4_-intoxicated rats with LCM at low (200 mg/kg bw) and high doses (400 mg/kg bw) markedly (*p* < 0.05) reduced the expression levels of the above genes. However, treatment with LCM alone showed non-significant (*p* > 0.05) differences in the fold changes of these genes compared to control rats.

The expression level of GCLC, an antioxidant enzyme, was significantly (*p* < 0.05) elevated with the administration of CCl_4_ to rats compared to the control group. The expression level of GCLC was further enhanced by the co-administration of silymarin and LCM at both doses, comparable to that of the CCl_4_ treated group. However, there was no significant (*p* > 0.05) difference in the fold change of GCLC in rats treated with CCl_4_ + LCM (200 mg/kg) and CCl_4_ + LCM (400 mg/kg bw). The expression of GCLC was also enhanced (*p* < 0.05) in rats treated with LCM at low and high doses alone compared to those of control and CCl_4_-treated rats (Figure 3a, Table 8).

The results showed significantly (*p* < 0.05) enhanced expression of pro-inflammatory cytokines (IL-6, TNF-α) and chemokines (MCP-1) in CCl_4_-treated rats. Co-administration of LCM at low (200 mg/kg bw) and high (400 mg/kg bw) doses along with CCl_4_ also showed marked elevation in the expression of these genes compared to the control group. However, administration of LCM alone at low and high doses regulated the expression of these genes to levels close to the control group (Table 8). The gel electrophoresis of these genes indicated the corresponding regulation of genes as manifested in the RT-PCR analysis (Figure 3b).

### 2.8. Effect of LCM on Histoarchitecture of the Liver

**Figure 4:**
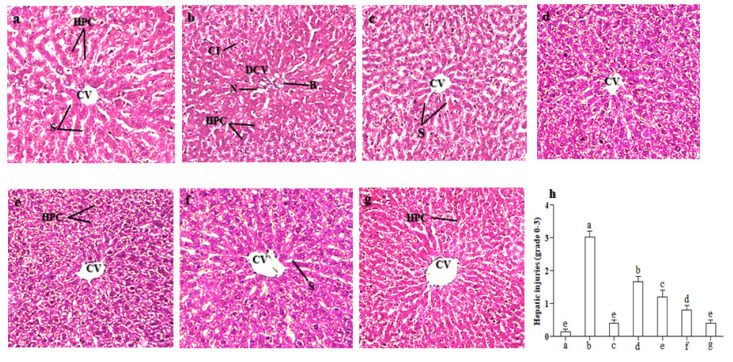
Protective potential of LCM on liver histopathology of rats (40× magnification with hematoxylin–eosin stain). (**a**) Control group, (**b**) CCl_4_ (1 mL/kg bw), (**c**) CCl_4_ + silymarin (200 mg/kg), (**d**) CCl_4_ + LCM (200 mg/kg), (**e**) CCl_4_ + LCM (400 mg/kg), (**f**) LCM (200 mg/kg), (**g**) LCM (400 mg/kg), (**h**) hepatic injuries ranging from 0 to 3, bars not sharing identical letters are significantly different at *p* < 0.05. CV; central vein. HPC; hepatocytes. DCV; damaged central vein. N; necrosis. B; ballooning of hepatocytes. LCM; *Linum corymbulosum* methanol extract.

Hepatic tissues of control rats exhibited normal histopathology of hepatocytes with vibrant central vein, sinusoids and Kupffer cells, as shown in Figure 4a. Administration of CCl_4_ to rats caused disrupted central veins, leukocyte infiltration, cellular hypertrophy and sinusoidal congestion (Figure 4b). The histopathological alterations induced by CCl_4_ were reduced and normal liver morphology was visible following co-administration of a reference drug, i.e., silymarin (Figure 4c). Similarly, hepatocellular injuries were decreased by co-administration of LCM at low (200 mg/kg bw) and high (400 mg/kg bw) doses to CCl_4_-intoxicated rats (Figure 4d,e). The typical conformation of the liver remained conserved with the treatment of LCM at low and high doses alone to rat (Figure 4f,g).

## 3. Discussion

Exposure to xenobiotics induces liver dysfunction that afterwards results in ER stress, inflammation and fibrosis. To overcome these damaging effects, several natural antioxidants have been reported recently that are beneficial for the human body [16]. Plants having polyphenols and flavonoids show a varied range of biological effects such as antioxidant, anti-inflammatory, anticancer, hepatoprotective and antidiabetic effects [17]. Oxidative stress provoked by CCl_4_, a reliable model for the evaluation of hepatic toxicity, was detected in hepatic injury in animals that have undergone CCl_4_ administration [18]. This ensues because active metabolites produced from CCl_4_ cause changes in mitochondrial permeability, cellular homeostasis and endoplasmic reticulum sequestration, resulting in cellular impairment [19]. Our study demonstrates that CCl_4_ administration for four weeks resulted in the development of inflammation as well as hepatic impairment in rats. Our outcomes are concomitant with the results of [20,21]. Furthermore, we tried to evaluate the hepatoprotective potential of *L. corymbulosum* methanol extracts and whether these plants diminish ER stress and the accompanying inflammation in rat liver tissues.

Phenolics and flavonoids can be easily identified and quantified with the use of HPLC techniques. This all has become possible due to the coupling of HPLC with that of DAD [HPLC–DAD), in which different signal wavelengths are used to confirm the presence of specific standard compounds. A similar protocol was followed by Zu et al. [22] to quantify catechin, kaempferol, rutin, isorhamnetin and quercetin. The selection of polyphenol standards was based on their reported medicinal properties, such as anticancer and antioxidant properties possessed by gallic acid and catechin. Catechin also protects against inflammation induced by free radicals and the resulting apoptosis [23]. Caffeic acid is known to have anticancer action [24]. Rutin is also famous for its antiviral, antioxidant, antiplatelet, hepatoprotective and antihypertensive properties [25]. Myricetin, another renowned flavonoid chelates ROS-producing metal ions and increases the potential of additional antioxidants. It is well known for having antidiabetic, anti-inflammatory, antiviral, anticancer, antithrombotic and anti-apoptotic effects and to prevent neurodegeneration [26]. Apigenin is known as a potent antioxidant, and anti-inflammatory agent [27] and also exerts antiviral, antimutagenic and purgative effects [28]. HPLC analysis of *L. corymbulsom* depicted the existence of rutin, catechin, myrecitin, apigenin and caffeic acid in the crude extract. The presence of these polyphenols in *L. corymbulosum* accounts for its antioxidant, anticancer and hepatoprotective properties.

Body weight is considered a substantial indicator for susceptibility to xenobiotics as well as a checkpoint for toxicity analysis [29]. A decrease in body weight of rats may be associated with the toxicity of CCl_4_ because it impairs the uptake and consumption of nutrients owing to malabsorption or maldigestion caused by gastrointestinal instabilities [30]. However, an increase in liver weight might be attributed to the accumulation of collagen [31] and lipids [32] which ultimately leads to a rise in the ratio of liver weight to body weight in rats treated with CCl_4_ compared to the control. In our present study, CCl_4_ treatment caused a reduction in percent increase in body weight, whereas absolute and relative liver weight was elevated. Our outcomes are in agreement with the findings of Lee et al. [33] who also described a reduction in percent increase in body weight and a rise in the absolute and relative weight of the liver in rats. However, treatment of rats with LCM ameliorated the toxicity instigated by CCl_4_ and reinstated the percent rise in body weight much closer to the control group. Similarly, absolute and relative liver weight was reduced with co-administration of LCM to CCl_4_-treated rats. These outcomes are in accord with other studies in which plant extracts were capable of increasing body weight and decreasing liver weight in rats administered with CCl_4_ [34]. Therapy with plant extracts restores the organ weight towards normal levels by diminishing the toxicity induced by CCl_4_ which is suggestive of the presence of protective ingredients in the extract.

The liver has been designated a supremely elusive organ concerning peroxidative impairments as it is rich in oxidizable elements. Metabolism of CCl_4_ in the liver causes lipid peroxidation, hepatocyte necrosis and demolition of membranes with ensuing leakage of essential enzymes into the bloodstream [2]. The first clue about hepatic impairment was found by appraisal of AST, ALP and ALT which are commonly used to evaluate plasma-membrane integrity and are also considered markers for liver function. AST is found in both the cytoplasm and mitochondria while ALP is a potential endoplasmic reticulum marker [29]. Our current study showed that CCl_4_ administration distinctly elevated the enzyme levels suggesting oxidative stress caused structural harm in hepatocytes. Treatment with LCM along with CCl_4_ decreased the level of liver marker enzymes, i.e., it protected the liver against CCl_4_-induceddamage. Sajid et al. [25] and Ali et al. [35] also reported that treatment with crude extracts of medicinally important plants results in the restoration of hepatic marker levels after CCl_4_ toxicity. Furthermore, a marked reduction in serum albumin levels and escalation in total bilirubin levels was also observed in this study. Their normal levels are mandatory for accurate liver physiology; a rise in bilirubin concentration is indicative of liver congestion. Administration of LCM at a high dose (400 mg/kg bw) to rats intoxicated with CCl_4_ exhibited more promising outcomes, reliably reinstating serum protein intensities close to the control group.

Nature has given us an antioxidant defense mechanism that includes both enzymatic, i.e., SOD, CAT and POD, and non-enzymatic antioxidants, those including carotenoids, tocopherols, glutathione, ascorbic acid and numerous others. These antioxidants protect against oxidative stress-induced injuries by scavenging ROS. Catalase (CAT) neutralizes H_2_O_2_ by peroxidation activity or through catalytic conversion. Superoxide dismutase, also known as metalloenzyme, converts superoxide radicals to H_2_O and O_2_ which are less toxic. Glutathione peroxidase shields against ROS-prompted toxicity by neutralizing peroxides through a catalytic reduction [36]. GSH is an important non-enzymatic radical scavenger that maintains intracellular redox homeostasis. It serves as an unspecific target for deleterious electrophilic metabolites; thereby acting as a critical marker for tissue liability against oxidative mutilation. A reduced level of GSH is suggestive of the initiation of liver necrosis [37]. In our study, the levels of intracellular GSH and antioxidant enzymes were rigorously depleted after CCl_4_ administration that caused accumulation of ROS ultimately leading to hepatocellular injury. Administration of LCM separately and in combination with CCl_4_ evidently restored antioxidant enzyme levels towards the control. The rise in GSH concentration may be because of de novo synthesis or due to GSH regeneration. Our study supports the fact that LCM possesses active constituents that are proficient in fighting against ROS-generated impairments by elevating the levels of these antioxidants. Our results are consistent with the work of El-Sayed et al. [36] who also reported a reduction in antioxidant levels in the disease state and restoration to normal state after treatment with plant extracts.

Nitric oxide plays a crucial role in oxidative stress; its elevated levels have been associated with oxidative damage through the formation of lipid peroxides and the generation of nitrite free radicals which are capable of inducing inflammation. Nitric oxide is produced from endothelial cells, Kupffer cells and hepatocytes in response to inflammation and tissue injury prompted by xenobiotics such as CCl_4_ [18]. Our current study displayed that the level of nitrites was significantly raised in rats administered with CCl_4_. This rise was distinctly lessened after administration with plant extract (LCM) suggesting an anti-inflammatory potential of the plant. The elevation in levels of TBARS, a product of lipid peroxidation in membranes, is another criterion for monitoring CCl_4_-prompted hepatic damage in rats. Chloride (Cl^−^) ions produced during the metabolism of CCl_4_ react with PUFA (polyunsaturated fatty acids), thereby prompting lipid peroxidation. It results in the production of TBARS which are reactive aldehydes responsible for hepatic injuries [38]. In our study, high levels of nitrite free radicals, H_2_O_2_ and TBARS and low levels of total protein were observed in animals treated with CCl_4_. Co-administration of plant extracts to CCl_4_-treated animals revealed a decline in the levels of these toxic entities and an elevation in tissue protein. However, treatment with extracts only restored these contents towards the levels in the control group. The decrease in TBARS might be attributed to the improved concentration of antioxidant enzymes which are capable of detoxifying these detrimental entities. These results are supported by previous work reported by Suzek et al. [39]. The potent antioxidant and curative potential of crude methanolic extract might be attributed to the existence of polyphenols including rutin, catechin and myricetin, as detected using HPLC analysis. In addition, gallic acid and apigenin are also found in LCM. All of these are well known for scavenging free radicals and thus curing disorders.

Anomalies in ER function critically affect the excretion of apolipoproteins. Earlier studies revealed the significance of proteins for the resilience of the liver against oxidative stress prompted by CCl_4_ [40]. Hepatocytes contain both smooth and rough endoplasmic reticulum which are required to accomplish several metabolic functions including plasma protein synthesis and secretion, cholesterol biosynthesis, lipoprotein secretion, most significantly VLDL (very low density lipoproteins), as well as xenobiotic metabolism [41]. Exposure to xenobiotics such as CCl_4_ results in the production of reactive oxygen entities that interrupt protein synthesis and protein folding by the endoplasmic reticulum. During the typical protein synthesis process in the ER, molecular chaperones are responsible for the correct folding of freshly produced proteins and the degradation of misfolded proteins. The unfolded protein response (UPR), a protective cellular response, is induced by the amassing of unfolded or misfolded proteins. It results in the reduction in abnormal protein levels by activation of several signaling pathways that halt protein translation and increase the production of important chaperons. However, if the amount of misfolded proteins disturbs this response, ER stress is provoked that activates caspases, ultimately leading to apoptosis [42]. A binding immunoglobulin protein (Bip), commonly known as GRP78, is the main regulator of ER stress since it binds to stress sensors in the lumen of the ER and inactivates them. Our current study revealed that the expression of GRP78 is enhanced manifold in animals intoxicated with CCl_4_. However, treatment with *L. corymbulosum* methanol extracts gradually reduced its expression and regulated its level comparable to the control. Our results are concomitant with the outcomes of Lee et al. [43] who also reported increased expression of XBP-1 and GRP78 due to swelling in the rough endoplasmic reticulum in hepatocytes caused by CCl_4_. GRP78 activates IRE-1α (inositol requiring enzyme 1α) by autophosphorylation which further prompts the production of a transcription factor XBP-1s by splicing an intron of XBP-1. These XBP-1s, after translocation to the nucleus, act with numerous co-regulators, thereby directing the expression of several genes involved in the ERAD (ER-associated degradation) [41]. CCl_4_-prompted ER stress reliably enhanced expression of XBP-1s manifold, with repression detected in animals receiving CCl_4_ and the plant extracts in a dose-reliant manner. However, the expression of XBP-1s was regulated normally in the animal group receiving the extract only, signifying that the polyphenols identified in the extracts might be responsible for the regulation of homeostasis in the endoplasmic reticulum and normalization of gene expression. Our study also focused on the mRNA expression of GCLC which is a crucial modulator of GSH. Its expression was also enhanced in animals receiving LCM suggesting that the extract enhanced the level of antioxidants. The UPR pathway includes another essential transmembrane protein, i.e., PERK (PKR-like ER protein kinase) which regulates ER stress by reducing protein contents and inhibiting cell cycle progression. This translational block mediated by PERK is indispensable for the production of JNK, NF-κB, TNF-α and IL-6 [41].

As a result of ER stress prompted by prolonged exposure to CCl_4_, misfolded proteins accumulate intracellularly, resulting in diseases related to cellular injury and inflammatory responses [42]. Pro-inflammatory cytokines including TNF-α and IL-6 and the chemokine MCP-1 play a crucial role in hepatic inflammation: MCP-1 is responsible for the recruitment of blood cells to the site of injury. A recent study showed increased expression of TNF-α, IL-6 and MCP-1 in animals who received CCl_4_, thus providing evidence of inflammation. Many earlier studies have reported these cytokines and chemokines to be involved in hepatic fibrosis [2,44,45]. However, treatment with LCM normalized the expression of these important genes in dose-dependent manner. Our study is in agreement with the work of Khalifa et al. [46] who also reported a strong anti-inflammatory potential of flavonoids in natural plant extracts.

The histopathology of hepatic tissues gives a visual impression for estimating CCl_4_-inducedtissue damage and imputes to our extract a protective aptitude in reversing CCl_4_-prompted noxious effects. The histological analysis of rat hepatic tissues treated with CCl_4_ displayed huge disruptions such as cellular hypertrophy, central lobule disruption, intrusion of inflammatory cells and congestion of sinusoids. These impairments were amended by co-administration of plant extracts at various doses with CCl_4_. However, treatment with extracts alone did not result in any change in liver morphology. Chen et al. [20] also conveyed similar outcomes when they reported *Schisandra lignin* extract to have protective potential against CCl_4_-prompted liver injury.

## 4. Materials and Methods

### 4.1. Plant Collection

The collection of *L. corymbulosum* (whole plant) was carried out at Quaid-i-Azam University, Islamabad, Pakistan in the month of March. Identification of *L. corymbulosum* was performed by Dr. Raees Khan and Dr. Mushtaq Ahmed, Department of Plant Sciences, Quaid-i-Azam University, Islamabad, Pakistan. A voucher sample of *L. corymbulosum* acquired the accession No. 129704 and was submitted to Pakistan Herbarium of Pakistan, Quaid-i-Azam University, Islamabad, Pakistan.

### 4.2. Preparation of Extracts

After collection and identification of plants, samples were cleaned and air-dried in an aerated but shaded area until complete removal of moisture. During drying, no microbial fermentation was observed. An electric grinder (60-mesh topology, Willy Mill) was used to powder the plant material and then these plant samples were utilized for solvent extraction. Dry powder of *L. corymbulosum* (500 g) was drenched in 2 L of methanol (95%) solution for 72 h at 25 °C and extracted thrice. Filtration was achieved through Whatman number 1 filter paper to gain refined methanolic plant extract. The filtrate was separated and then dried by using a rotary evaporator. Partial refining or parting of crude methanolic extract was carried out through solvent–solvent extraction.

### 4.3. High Performance Liquid Chromatography (HPLC–DAD) Analysis

HPLC analyses of crude extract of *L. corymbulosum* and selected plant fractions were accomplished employing HPLC–DAD (Agilent 1200, Waldbronn, Germany) equipped with a Zorbex RXC8 (Agilent, Santa Clara, CA, USA) analytical column with 5 μm particle size and 25 mL capacity utilizing an earlier described method [47]. The mobile phase comprised eluent A (acetonitrile–methanol–water-acetic acid/5:010:85:01) and eluent B (acetonitrile–methanol–acetic acid/40:060:61). The gradient (A:B) employed was the following: 0–20 min (0 to 50% B), 21–25 min (50 to 100% B), 26–30 min (100% B) and 31–40 (100 to 0% B) at a flow rate of 1 mL/min. The samples and standards were prepared in HPLC-grade methanol (1 mg/mL), filtered using a 0.456 μm membrane filter and 20 μL was inoculated for examination. Amongst the standards rutin was assessed at 257 nm, gallic acid and catechin at 279 nm, apigenin and caffeic acid at0325 nm, while myricetin, kaempferol and quercetin were investigated at 368 nm [48]. The analysis was executed in triplicate for 10 min each, and the column was cleaned after every run. By peak integration using an external standard method, quantification was performed.

### 4.4. Animals

The projected outcomes were studied in seven weeks old Sprague Dawley rats having a body weight between 170 and 200 g. Forty-nine female and forty-nine male rats were acquired from the primate facility, Quaid-i-Azam University, Islamabad, Pakistan. These rats were kept in a ventilated room with proper food and water. Prior to the research investigation, ethical approval # Bch-0362 was obtained from the Ethics Committee, Quaid-i-Azam University, Islamabad.

### 4.5. Acute Toxicity Assessment

The acute toxicity of the extract was assessed according to the guidelines 425 advocated by the Organization for Economic Cooperation and Development. The Sprague Dawley female rats (n = 5) were used for the assessment of acute toxicity. These were kept in fasting conditions overnight. The extract mockups were prepared in DMSO (10%). Initially, a dosage of 50 mg/kg body.weight was administered intra-gastrically to animals and these were kept under examination for determination of mortality rate and other parameters for a duration of about 2 weeks. Since no toxicity progression was observed, the method was followed with a greater dosage of plant extracts, i.e., 100, 200, 400, 1000, 2000, 3000 and 4000 mg/kg body weight administered to five female rats (for each respective dose). Unusual behavior and mortality were not detected even at the highest dosage of 4000 mg/kg body weight after two weeks of drug administration. Therefore one-tenth (400 mg/body weight) and one-twentieth (200 mg/kg body weight) of the highest dosage were carefully elected for evaluation of hepatoprotective influence against CCl4-prompted oxidative stress [49]. The blood of the treated rats with 2000 mg/kg bw and 4000 mg/kg bw was used for hematological investigation [50].

### 4.6. Experimental Design

The hepatoprotective potential of LCM was analyzed using 49 Sprague Dawley male rats divided into seven different groups each containing seven rats. In each rat, 1 mL/kg bw of CCl_4_ (Sigma-Aldrich) diluted in olive oil (CCl_4_:olive oil; 3:7 *v*/*v*) was intraperitoneally injected [51] for 4 weeks, while the methanolic extract of the plants and the standard hepatoprotective drug silymarin were orally administered by using feeding tubes. Silymarin is a natural compound that is widely utilized in traditional medicine and has been investigated in formal scientific studies [52].

### 4.7. Dose Regimen

Group1 (Control): No treatment

Group2 (CCl_4_): 1 mL/kg bw of CCl_4_ (30% CCl_4_ in olive oil)

Group3 (CCl_4_ + silymarin): 30% CCl_4_ + 200 mg/kg of Silymarin

Group4 (CCl_4_ + LCM low dose): 30% CCl_4_ + 200 mg/kg of LCM

Group5 (CCl_4_ + LCM high dose): 30% CCl_4_ + 400 mg/kg of LCM

Group6 (LCM—low dose): 200 mg/kg LCM

Group7 (LCM—high dose): 400 mg/kg LCM

Upon dosage completion, the rats were subjected to dissection. Before dissection without any treatment normal feed was provided to all the rats for 24 h. Chloroform anesthesia was administered, and rats were dissected on the ventral side of the body. The cardiac puncture was performed by using 23-G1 syringes and the blood was collected in vacutainers. The next step was centrifugation of tubes at 8000 rpm at 40 °C for 15 min and serum was collected from every tube for biochemical analysis. The liver tissues were removed and rinsed with saline (ice-cold) to get rid of debris and then kept at −70 °C in liquid nitrogen until tissue homogenate assessments. For histopathology testing, some parts of the liver were stored in 10% formalin buffered with phosphate at room temperature for 48 h [53].

### 4.8. Body Weight and Organ Weight

Rats from all groups were weighed on the first and final day of the experiment. The initial and final body weight of each rat were compared to estimate the increase in body weight percentage. During the dissection process, liver tissues were removed and placed in saline. After this, relative liver weight was observed for each rat in the different groups.

### 4.9. Biochemical Studies of Serum

The levels of various serum markers comprising alkaline phosphatase (ALP), aspartate transaminase (AST), alanine transaminase (ALT), total bilirubin and albumin were estimated through liver function tests utilizing the standard manual of AMP diagnostic kits Krengasse, Graz, Austria).

### 4.10. Biochemical Studies of Tissues

500 mg of liver tissues were weighed and homogenized in 100 mM of 1 mL of potassium phosphate buffer along with 1 mM of EDTA and pH was maintained at 7.4. Then the homogenates were centrifuged at 4 °C at 12,000× *g* for 20 min [50]. After centrifugation, the supernatant obtained was utilized for the execution of the following assays.

#### 4.10.1. Catalase Assay (CAT)

CAT activity was assessed on the grounds of decomposition of H_2_O_2_ by following the procedure previously reported by [54]. To 100 μL supernatant of the homogenate, we added 400 μL H_2_O_2_ (5.9 mM), and 2500 μL PBS (50 mM) and mixed them all together. An alteration in the optical density of the mixture was observed at 520 nm after 1 min. One unit CAT activity means a change in absorbance of 0.01 units/min.

#### 4.10.2. Peroxidase Assay (POD)

Guaiacol peroxidation was a criterion to assess the potential of the peroxidase enzyme [55]. To evaluate supernatant POD activity, the reaction mix consisted of 600 µL of 40 mM H_2_O_2_, 300 µL of 20 mM guaiacol, 3500 µL of 50 mM PBS (pH 5) and 2000 µL of the supernatant. Absorbance was recorded at 470 nm, after one-minute intervals. Variation in absorbance of 0.01 units/min is equal to one unit POD activity.

#### 4.10.3. Superoxide Dismutase Assay (SOD)

The practice of Misra and Fridovich [56] was followed to determine SOD activity. The reaction blend of this test consists of 186 mM phenazine methosulphate (0.1 mL), 0.052 mM Na_4_P_2_O_7_ buffer (1.2 mL) and 0.3 mL of supernatant. Centrifugation of this mixture was executed at 1500× *g* for 10 min and then again at 10,000× *g* for 15 min. The enzymatic reaction of this mixture was started after adding 0.2 mL of 780 μM NADH. The absorbance was determined at 560 nm and the outcomes were assessed in units/mg protein.

#### 4.10.4. Reduced Glutathione Assay (GSH)

The level of GSH was measured by using the methodology of Iqbal and Wright [57] with some modifications. Firstly, 1000 µL of homogenate mixture was taken and dissolved in 1000 µL of 4% sulfosalicylic acid. Then this solution was incubated at 4 °C for one hour and centrifugation was executed at 40 °C for 20 min at 1200× *g*. Following that, 100 µL of supernatant was poured into 2.7 mL of PBS (pH 7.4). Then 200 µL of DTNB (100 mM) was added to it. The reaction between GSH and DTNB produced yellow-colored GSH. The absorbance was measured using a spectrophotometer at 412 nm.

#### 4.10.5. Estimation of Lipid Peroxidation Assay (TBARS)

Iqbal and Wright [57] reported a method for performing a lipid peroxidation assay with some amendments. According to their protocol, a reaction mixture was made which contained 580 µL of 0.1 M PBS (pH 7.4), 200 µL homogenate sample, 200 µL of 100 mM ascorbic acid and 20 µL of FeCl_3_ (100 mM) making a total solution volume of 1000 µL. Using a shaker water bath, the reaction mixture was incubated for 1 h at 37 °C. Then the reaction was stopped with 1000 µL TCA (10%). Later on, all tubes were placed in boiling H_2_O. After that, 1000 µL TBA (0.67%) was mixed in and the reaction tubes were kept on crushed ice for twenty minutes. The tubes were then centrifuged for 10 min at 2500× *g*. Finally, the amount of TBARS in all samples was measured by taking absorbance against a blank reagent at 535 nm using a spectrophotometer. The outcomes were calculated as nM TBARS/minute/mg of tissues at 37 °C, using a molar extinction coefficient of 1.56 × 10^5^/M/cm.

#### 4.10.6. Hydrogen Peroxide Assay (H_2_O_2_)

Pick and Keisari [58] presented a protocol for H_2_O_2_-level determination based on the principle of hydrogen peroxide intervening with horseradish-peroxidase-reliant oxidation of phenol red. According to this procedure, the reaction mixture comprised 3 ml of tissue homogenate, which was dissolved in 1000 µL of 0.28 nM phenol red, dextrose (6.6 nM), horseradish peroxidase and 0.05 M PBS (pH 8.0), and incubated for 60 min at 37 °C temperature. Then, 10 µL of 10 N sodium hydroxide was poured to stop the reaction. After that, centrifugation of the solution was carried out for five minutes at 800× *g*. In the end, absorbance was taken against a blank reagent at 615 nm on a spectrophotometer. The hydrogen peroxide amount formed was defined as nM H_2_O_2_ per min per milligram tissue by comparison to a hydrogen peroxide-oxidized phenol red standard curve.

#### 4.10.7. Nitrite Assay

Griess reagent was utilized for the evaluation of nitrite assay by using the procedure of Majid et al. [59]. Homogenization of hepatic tissues was achieved with equal volumes of NaOH and ZnSO_4._ The master mix comprised 1 mL of tissue homogenate which was dissolved in 3 M of 100 µL NaOH and 5% ZnSO_4_ solution. After that, centrifugation was carried out for 15–20 min at 6400× *g*. Then 20 µL of supernatant was dissolved in 1 mL Griess reagent. Color variations were observed and absorbance was measured at 540 nm. A sodium nitrite standard curve was utilized to estimate nitrite concentration in the tissue sample.

#### 4.10.8. Tissue Protein Assessment

Assessment of total protein within tissues was performed by using the practice of Bensadoun and Weinstein [60]. For this, 80 mg organ was homogenized using potassium phosphate buffer. This reaction mix was centrifuged for 20 min at 10,000× *g* at 4 °C temperature to get the supernatant. Then, 0.1 mL of supernatant was dissolved in 1 mL of alkaline solution and incubated for 10 min. Folin–Ciocalteu’s phenol reagent (1:1) was added in a similar proportion and mixed carefully using a vortex machine. Samples were incubated for 30 min at 25 °C. Afterwards, absorbance was recorded at a wavelength of 595 nm. Total solubilized protein was calculated with a BSA standard curve.

### 4.11. Histopathological Studies

For the assessment of the histopathology of the liver, paraffin fixed slices were used. A multi-step methodology was used to fix the testing samples and avert tissue decay and conserve the morphology of tissues. For that purpose, 10% formalin was used to preserve fresh tissues of the liver. Variable concentrations of alcohol such as 50%, 70%, 90% and 100% were applied to wash the fixed tissues. The main objective of this step was to fix the tissue on a rigid solid matrix that makes it easy to cut thin slices of tissue. The last step was the preparation of slides by segmenting 4–5 µm thin layers of fixed tissue samples and then hematoxylin and eosin staining was carried out. The slides were examined to analyze the protective potential of LCM against CCl_4_-intoxicated liver and results were interpreted using the ordinal histological scoring system by adding the scores (0–3) of steatosis, leukocyte infiltration, fatty degeneration, perivascular and lobular necrosis, cellular hypertrophy and sinusoidal obstruction [61]. The ordinal scoring system depicts 0 for normal, 1 for mild, 2 for moderate and 3 for severe aberrations. Using a light microscope (DIALUX 20EB) at 40× magnification, slides of experimental groups were photographed and studied.

### 4.12. RNA Extraction

RNA was extracted using the TRIzol method [62]. A total of 1 mL TRIzol/80 mg was added and homogenization was performed. After this, ice-cold chloroform was added to the mixture and incubated for 10 min. Centrifugation was carried out at 10,000× *g* at 4 °C for 15 min. After centrifugation, three visible layers appeared; a top-layer aqueous phase (comprising RNA), an intermediate layer, and an organic bottom phase (comprising DNA and proteins). The supernatant was collected and transferred into a new Eppendorf tube. After that, chilled isopropanol was added for the precipitation of RNA from the upper layer. With the help of a pipette, much of the remaining isopropanol was removed, taking extra care not to touch the RNA pellet. Washing was performed with 75% ethanol. After that, 1 mL of extra-pure ethanol (100%) was added and centrifugation was performed for 5 min at 7500× *g*. The RNA pellet was liquefied in 30–50 µL of RNAse free water of molecular grade and the tubes were retained on crushed ice. RNA could then be preserved at −80 °C.

### 4.13. RT-PCR Analysis

DNase-I was utilized for the elimination of genomic DNA contagion from the total RNA. A reverse transcription scheme was employed to synthesize first strand cDNAs at 42 °C for fifty minutes, 99 °C for five minutes and 4 °C for five minutes in a Thermal Gene Cycler (Biometra, Göttingen, Germany). The real-time polymerase chain reaction was performed with a 25 μL reaction mix containing 12.5 μL of 2x Maxima SYBR Green/ROX qPCR master mix (Thermo Scientific, Waltham, MA, USA), 0.3 μM primers and the cDNAs. The settings of the PCR reaction were as follows: 95 °C for ten minutes, 40 cycles of 95 °C for 15 s, 60 °C for 30 s and 72 °C for 30 s. Melting curves were evaluated by utilizing heat-denaturing-PCR products over 35 °C ascending at 0.2 °C/s from 60 to 95 °C. β-actin was utilized as an internal control. The sequence of primers for GRP78, GCLC, XBPS, XBPT, XBPU, MCP-1, IL-6, TNF- α and β-actin were prepared based on the rat repeated cDNA sequences. The sequences of primers for the different genes are given in Table 9. The outcomes were also verified using agarose gel-electrophoresis with RT-PCR products.

### 4.14. Relative Quantification of Gene Expression

Relative quantification of various genes was determined using a comparative CT method by using the β-actin gene as an internal control. For this purpose, ∆CT of the treated samples (CT of treated–CT of internal control) and ∆CT of the control sample (CT of control–CT of internal control) for the gene of interest was calculated. Then ∆∆CT = ∆CT (treated)–∆CT (control) was determined. CT was used to determine fold change due to treatment = 2^−∆∆CT^ [63]. *p* values below 0.05 were considered significant.

### 4.15. Statistical Analysis

The experimental data were articulated as mean ± SD (standard deviation). Graphical interpretation for gene expression analysis was performed using GraphPad prism 5. The differences between various groups under study were calculated using one-way analysis of variance (ANOVA) using the software Statistix 8.1. Statistical significance for the validation of inter-group comparison was assumed at *p*-values ≤ 0.05.

## 5. Conclusions

The current study indicated that *L. corymbulosum* possesses potent antioxidant and anti-inflammatory activity. Results demonstrated a reduction in ROS which is perhaps due to the occurrence of polyphenolics in the plant. Therefore, it can be concluded that these natural phytochemicals are involved in the amelioration of inflammation. They also suggested that the plant could be a potent hepatoprotective agent. Our data depicted that the plant has the aptitude to normalize the actions of antioxidant enzymes, mRNA expression of ER stress indicators and inflammatory intermediaries, thereby efficiently preserving the physiology and morphology of hepatic tissue and emerging as a promising contender for drug development against several liver impairments.

## Figures and Tables

**Table 1:** Retention time, optimized wavelength and regression analysis of reference flavonoids and phenolics for *L. corymbulosum* methanol extract and derived fractions.

Reference Flavonoids/Phenolics	Signal Wavelength	Retention Time (min)	Regression Analysis	R^2^
Rutin	257	13.874	y = 8.336x + 22.21	0.9899
Gallic acid	279	4.131	y = 24.85x − 47.147	0.9928
Catechin	279	7.991	y = 7.987x − 16.913	0.9967
Caffeic acid	325	10.336	y = 10.24x + 26.27	0.9875
Apigenin	325	22.583	y = 12.53x + 77.54	0.9854
Myricetin	368	15.310	y = 2.04x − 10.16	0.9897
Quercetin	368	19.052	y = 12.21x − 20.34	0.9993
Kaempferol	368	21.668	y = 8.78x + 120.24	0.9994

**Table 2:** HPLC–DAD analysis of *L. corymbulosum* methanol extract and its derived fractions.

Extracts	Polyphenolics (μg/mg of Extract)							
	Rutin	Catechin	Gallic Acid	Caffeic Acid	Myricetin	Apigenin	Quercetin	Kaempferol
LCM	1.92	9.94	-	7.38	3.37	2.64	-	-
LCE	4.71	-	-	6.21	-	-	-	-
LCB	51.48	43.7	3.11	1.43	21.82	-	-	-
LCA	-	7.97	6.11	6.4	-	-	-	-

LCM; *L. corymbulosum* methanol extract. LCE; *L. corymbulosum* ethyl acetate fraction. LCB; *L. corymbulosum* butanol fraction. LCA; *L. corymbulosum* aqueous fraction.

**Table 3:** Effect of different treatments with *L. corymbulosum* on hematological parameters.

Treatment Groups	WBC (10^3^/mm^3^)	RBC (10^6^/mm^3^)	HGB (g/dL)	MCV (fL)	PLT (10^3^/mL)	LYM (10^3^/mm^3^)	MCHC (g/dL)
**Control**	6.82 ± 0.1 ^b^	7.33 ± 0.08 ^ab^	13.78 ± 0.07 ^a^	42.36 ± 0.30 ^c^	170.4 ± 1.0 ^b^	5.24 ± 0.12 ^c^	34.5 ± 0.92 ^b^
**LCM (2000 mg/kg)**	12.67 ± 0.26 ^a^	7.10 ± 0.12 ^b^	13.3± 0.15 ^a^	47.44 ± 0.83 ^b^	159.1 ± 1.31 ^c^	7.50 ± 0.37 ^b^	37.7 ± 0.55 ^a^
**LCM (4000 mg/kg)**	13.13 ± 0.35 ^a^	7.57 ± 0.23 ^a^	14.3 ± 0.69 ^a^	51.6 ± 1.05 ^a^	186.23 ± 1.20 ^a^	9.75 ± 0.22 ^a^	39.2 ± 0.52 ^a^

Values are given as mean ± SD (n = 7). Means with different superscript letters ^(a–c)^ in a column specify significance at *p* < 0.05. LCM; *Linum corymbulosum* methanol extract. WBC; white blood cells. RBC; red blood cells. HGB; hemoglobin. MCV; mean corpuscular volume. PLT; platelets. MCHC; mean corpuscular hemoglobin concentration.

**Table 4:** Effect of different treatments with *L. corymbulosum* on % increase in body weight and organ weight of rat.

Treatment Groups	Initial Body Weight (g)	Final Body Weight (g)	% Increase	Absolute Liver Weight (g)	Relative Liver Weight (mg/g)
Control	153.9 ± 1.5	249.3 ± 2.0	61.95 ± 2.6 ^a^	7.65 ± 0.22 ^d^	30.69 ± 0.8 ^e^
CCl_4_ (1 mL/kg)	160.4 ± 1.8	213.3 ± 0.62	32.98 ± 1.3 ^e^	9.42 ± 0.04 ^a^	44.16 ± 0.2 ^a^
CCl_4_ + silymarin (200 mg/kg)	158.2 ± 1.3	247.3 ± 0.9	56.32 ± 1.8 ^b^	7.96 ± 0.13 ^d^	32.18 ± 0.6 ^e^
CCl_4_ + LCM (200 mg/kg)	159.6 ± 1.4	220.6 ± 0.6	38.23 ± 1.17 ^d^	8.98 ± 0.12 ^b^	40.74 ± 0.58 ^b^
CCl_4_ + LCM (400 mg/kg)	156.0 ± 1.4	221.7 ± 1.6	42.16 ± 2.0 ^cd^	8.5 ± 0.23 ^c^	38.33 ± 1.29 ^c^
LCM(200 mg/kg)	161.3 ± 1.2	237.6 ± 1.2	47.31 ± 1.8 ^c^	8.41 ± 0.11 ^c^	35.4 ± 0.54 ^d^
LCM(400 mg/kg)	161.4 ± 0.7	251.3 ± 1.4	55.72 ± 1.5 ^b^	8.07 ± 0.12 ^cd^	32.13 ± 0.28 ^e^

Values are given as mean ± SD (n = 7). Means with different superscript letters ^(a–e)^ in a column specify significance at *p* < 0.05. LCM; *Linum corymbulosum* methanol extract. CCl_4_; carbon tetrachloride.

**Table 5:** Effect of different treatments with *L. corymbulosum* on liver serum markers.

Treatment Groups	ALT (U/L)	AST (U/L)	ALP (U/L)	Albumin (mg/dL)	Total Bilirubin (mg/dL)
Control	44.43 ± 0.25 ^g^	57.13 ± 0.44 ^g^	66.76 ± 0.78 ^g^	4.76 ± 0.22 ^a^	0.45 ± 0.02 ^f^
CCl_4_ (1 mL/kg)	156.8 ± 0.37 ^a^	171.8 ± 1.11 ^a^	147.8 ± 1.07 ^a^	1.98 ± 0.08 ^f^	1.80 ± 0.02 ^a^
CCl_4_ + silymarin (200 mg/kg)	55.65 ± 0.43 ^e^	73.40 ± 0.74 ^f^	78.39 ± 0.46 ^f^	4.22 ± 0.10 ^b^	0.66 ± 0.04 ^e^
CCl_4_ + LCM (200 mg/kg)	111.8 ± 0.84 ^b^	131.2 ± 1.36 ^b^	120.4 ± 1.06 ^b^	2.57 ± 0.10 ^e^	1.19 ± 0.05 ^b^
CCl_4_ + LCM (400 mg/kg)	93.97 ± 0.41 ^c^	108.8 ± 0.65 ^c^	112.8 ± 0.76 ^c^	2.99 ± 0.12 ^d^	0.93 ± 0.02 ^c^
LCM (200 mg/kg)	53.38 ± 0.53 ^d^	98.11 ± 0.73 ^d^	98.02 ± 0.56 ^d^	3.78 ± 0.13 ^c^	0.82 ± 0.02 ^d^
LCM (400 mg/kg)	46.17 ± 0.72 ^f^	78.54 ± 0.96 ^e^	87.79 ± 0.66 ^e^	3.90 ± 0.06 ^c^	0.71 ± 0.04 ^e^

Values are given as mean ± SD (n = 7). Means with different superscript letters ^(a–g)^ in a column specify significance at *p* < 0.05. LCM; *Linum corymbulosum* methanol extract. CCl_4_; carbon tetrachloride.

**Table 6:** Effect of different treatments with *L. corymbulosum* on liver tissue antioxidant enzymes.

Treatment Groups	CAT (U/min)	POD (U/min)	SOD (U/mg Protein)	GSH (µmol/mg)
Control	9.82 ± 0.70 ^a^	12.11 ± 1.37 ^a^	4.78 ± 0.63 ^a^	17.05 ± 0.94 ^a^
CCl_4_ (1 mL/kg)	1.58 ± 0.37 ^f^	3.88 ± 0.35 ^f^	0.84 ± 0.18 ^e^	4.90 ± 0.45 ^f^
CCl_4_ + silymarin (200 mg/kg)	8.20 ± 0.68 ^b^	11.52 ± 1.5 ^ab^	3.57 ± 0.74 ^b^	14.04 ± 1.6 ^b^
CCl_4_ + LCM (200 mg/kg)	2.94 ± 0.32 ^e^	5.54 ± 0.62 ^e^	1.58 ± 0.18 ^de^	7.12 ± 0.62 ^e^
CCl_4_ + LCM (400 mg/kg)	4.46 ± 0.31 ^d^	7.22 ± 0.55 ^d^	1.94 ± 0.61 ^cd^	8.68 ± 0.45 ^d^
LCM (200 mg/kg)	6.9 ± 0.80 ^c^	8.84 ± 0.65 ^c^	2.74 ± 0.55 ^bc^	11.38 ± 0.90 ^c^
LCM (400 mg/kg)	7.54 ± 0.57 ^bc^	10.24 ± 0.42 ^bc^	3.47 ± 0.65 ^b^	13.47 ± 0.92 ^b^

Values are given as mean ± SD (n = 7). Means with different superscript letters ^(a–f)^ in a column specify significance at *p* < 0.05. LCM; *Linum corymbulosum* methanol extract. CCl_4_; carbon tetrachloride.

**Table 7:** Effect of *L. corymbulosum* on hepatic proteins, TBARS, H_2_O_2_ and nitrite content.

Treatment Groups	Protein (μg/mg Tissue)	TBARS (nM/min/mg Protein)	H_2_O_2_ (nM/min/mg Tissue)	Nitrite Content (μM/mL)
Control	11.47 ± 0.74 ^a^	41.40 ± 1.36 ^g^	5.70 ± 0.29 ^d^	57.80 ± 1.52 ^f^
CCl_4_ (1 mL/kg)	5.91 ± 0.31 ^f^	99.44 ± 2.04 ^a^	11.02 ± 0.55 ^a^	95.48 ± 3.06 ^a^
CCl_4_ + silymarin (200 mg/kg)	10.72 ± 0.67 ^a^	46.28 ± 1.84 ^f^	6.33 ± 0.38 ^d^	65.74 ± 2.09 ^e^
CCl_4_ + LCM (200 mg/kg)	6.86 ± 0.37 ^e^	86.86 ± 2.11 ^b^	9.39 ± 0.64 ^b^	87.88 ± 2.72 ^b^
CCl_4_ + LCM (400 mg/kg)	7.99 ± 0.31 ^d^	79.14 ± 2.77 ^c^	8.0 ± 0.31 ^c^	81.88 ± 1.12 ^c^
LCM (200 mg/kg)	8.86 ± 0.42 ^c^	63.42 ± 2.56 ^d^	7.89 ± 0.36 ^c^	77.67 ± 1.98 ^d^
LCM (400 mg/kg)	9.74 ± 0.36 ^b^	51.14 ± 2.12 ^e^	6.34 ± 0.35 ^d^	68.65 ± 2.0 ^e^

Values are given as mean ± SD (n = 7). Means with different superscript letters ^(a–g)^ in a column specify significance at *p* < 0.05. LCM; *Linum corymbulosum* methanol extract. CCl_4_; carbon tetrachloride.

**Table 8:** Effect of different treatments with *L. corymbulosum* on fold changes of various genes involved in CCl_4_-induced ER Stress.

Treatment Groups	GRP78	XBPt	XBPs	XBPu	GCLC	MCP-1	IL-6	TNF-ɑ
Control	1.0 ± 0.0 ^f^	1.0 ± 0.0 ^e^	1.0 ± 0.0 ^f^	1.0 ± 0.0 ^e^	1.0 ± 0.0 ^e^	1.0 ± 0.0 ^e^	1.0 ± 0.0 ^e^	1.0 ± 0.0 ^f^
CCl_4_	6.89 ± 0.17 ^a^	2.83 ± 0.10 ^a^	7.10 ± 0.13 ^a^	2.46 ± 0.10 ^a^	2.24 ± 0.10 ^d^	4.14 ± 0.09 ^a^	3.63 ± 0.12 ^a^	8.96 ± 0.18 ^a^
CCl_4_ + silymarin (200 mg/kg)	1.15 ± 0.13 ^ef^	0.96 ± 0.10 ^e^	1.18 ± 0.12 ^f^	1.27 ± 0.11 ^de^	3.66 ± 0.12 ^a^	1.23 ± 0.10 ^e^	0.98 ± 0.10 ^e^	1.39 ± 0.13 ^f^
CCl_4_ + LCM (200 mg/kg)	4.46 ± 0.18 ^b^	2.46 ± 0.09 ^b^	6.02 ± 0.18 ^b^	2.26 ± 0.13 ^a^	3.02 ± 0.11 ^b^	3.27 ± 0.11 ^b^	2.97 ± 0.08 ^b^	5.88 ± 0.15 ^b^
CCl_4_ + LCM (400 mg/kg)	3.38 ± 0.13 ^c^	1.93 ± 0.10 ^c^	4.76 ± 0.14 ^c^	1.73 ± 0.11 ^bc^	3.82 ± 0.12 ^c^	2.57 ± 0.10 ^c^	2.24 ± 0.10 ^c^	4.32 ± 0.17 ^c^
LCM (200 mg/kg)	2.14 ± 0.15 ^d^	1.55 ± 0.11 ^d^	2.99 ± 0.13 ^d^	1.82 ± 0.12 ^b^	2.34 ± 0.16 ^d^	1.70 ± 0.12 ^d^	1.67 ± 0.11 ^d^	2.53 ± 0.18 ^d^
LCM (400 mg/kg)	1.47 ± 0.07 ^e^	1.20 ± 0.09 ^e^	1.86 ± 0.13 ^e^	1.48 ± 0.08 ^cd^	2.67± 0.09 ^c^	1.58 ± 0.13 ^d^	1.24 ± 0.07 ^e^	1.91 ± 0.15 ^e^

Values are given as mean ± SD (n = 3). Means with different superscript letters ^(a–f)^ in a column specify significance at *p* < 0.05. LCM; *Linum corymbulosum* methanol extract. CCl_4_; carbon tetrachloride.

**Table 9:** Primer sequences for real-time PCR assay.

Gene		Primer Sequences (5′-3′)	Product Length (bp)
GRP78	Forward	GAAATTTCTGCTATGGTTCTCACT	292
	Reverse	GAAGTAAGCTGGTACAGTCACA	
GCLC	Forward	GTGGACACCCGATGCAGTAT	192
	Reverse	TCATCCACCTGGCAACAGTC	
XBP-1s	Forward	TGAGTCCGCAGCAGGTGCA	155
	Reverse	ACAGGGTCCAACTTGTCCAGAA	
XBP-1t	Forward	CCCTGGTTACTGAAGAGGTC	238
	Reverse	GTCCAACTTGTCCAGAATGC	
XBP-1u	Forward	AAAGCGCTGCGGAGGAAA	170
	Reverse	AGCTGGAGTTTCTGGTTCTCT	
TNF-ɑ	Forward	GCTCCCTCTCATCAGTTCCA	265
	Reverse	GGTTGTCTTTGAGATCCATGC	
IL-6	Forward	GTCAACTCCATCTGCCCTTC	126
	Reverse	ACTGGTCTGTTGTGGGTGGT	
MCP	Forward	CAAGATGTGCGCTGAGGACA	113
	Reverse	TTCTCTATTGGTGGCAGACC	
β-actin	Forward	CCTCTATGCCAACACAGTGC	178
	Reverse	CATCGTACTCCTGCTTGCTG	

## Data Availability

The data presented in this manuscript belong to the research work performed by Dr. Riffat Batool under the supervision of Dr. Muhammad Rashid Khan and has not been submitted/published elsewhere.

## Sample Availability

Not available.

## Abbreviations

GRP78, glucose regulated protein; XBP-1 t, x-box binding protein-1 total; XBP-1 s, x-box binding protein-1 spliced; XBP-1 u, x-box binding protein-1 unspliced; GCLC, glutamate–cysteine ligase catalytic subunit; TNF-α, tumor necrosis factor-α; IL-6, interleukin 6; MCP-1, monocyte chemo attractant protein-1; CAT, catalase; POD, peroxidase; SOD, superoxide dismutase; GSH, reduced glutathione.
